# Development of a nested-PCR assay for the rapid detection of *Pilidiella granati* in pomegranate fruit

**DOI:** 10.1038/srep40954

**Published:** 2017-01-20

**Authors:** Xue Yang, Uzma Hameed, Ai-Fang Zhang, Hao-Yu Zang, Chun-Yan Gu, Yu Chen, Yi-Liu Xu

**Affiliations:** 1 Institute of Plant Protection and Agro-products Safety, Anhui Academy of Agricultural Sciences, Hefei 230031, China; 2 Scientific Observing and Experimental Station of Crop Pests in Hefei, Ministry of Agriculture, China; 3 Laboratory of Quality and Safety Risk Assessment for Agro-Products (Hefei), Ministry of Agriculture, China; 4 Institute of Industrial Biotechnology, Government College University, Lahore 54000, Pakistan; 5 Anhui Academy of Agricultural Sciences, Hefei 230031, China; 6 Key Laboratory of Genetic Improvement and Ecophysiology of Horticultural Crop, Anhui Province, China

## Abstract

*Pilidiella granati*, a causal agent of twig blight and crown rot of pomegranate, is an emerging threat that may cause severe risk to the pomegranate industry in the future. Development of a rapid assay for the timely and accurate detection of *P. granati* will be helpful in the active surveillance and management of the disease caused by this pathogen. In this study, a nested PCR method was established for the detection of *P. granati*. Comparative analysis of genetic diversity within 5.8S rDNA internal transcribed spacer (ITS) sequences of *P. granati* and 21 other selected fungal species was performed to design species-specific primers (S1 and S2). This primer pair successfully amplified a 450 bp product exclusively from the genomic DNA of *P. granati*. The developed method can detect 10 pg genomic DNA of the pathogen in about 6 h. This technique was successfully applied to detect the natural infection of *P. granati* in the pomegranate fruit. The designed protocol is rapid and precise with a high degree of sensitivity.

Pomegranate (*Punica granatum*) is one of the most ancient and important economic fruit crops in the world with broad geological distribution. It is native to Iran and Turkey but has been cultivated throughout the Mediterranean region and northern India since ancient times^1^,^2^,^3^. Pomegranate fruit is rich in a variety of compounds such as alkaloids, flavonoids, anthocyanins, steroids, sterols, vitamin C, fatty acids, organic acids, tannins, and several resinous and polyphenolic substances. All these compounds bequeath the fruit with antioxidant, antimicrobial, anti-inflammatory, anti-carcinogenic, skin regeneration, and cardiovascular protection properties^2^,^3^,^4^,^5^,^6^. These remarkable health benefits have increased the off-season demand for both the pomegranate fruit and juice. Therefore, pomegranate cultivation and storage facilities all over the world are rapidly expanding^2^.

Pomegranate is susceptible to numerous pre- and post-harvest fungal diseases. The most prominent fungal pathogens of pomegranate include *Botrytis cinerea, Aspergillus niger, Penicillium* spp., *Alternaria* spp., *Trichoderma* spp., *Colletotrichum gloeosporioides, Pestalotia brevista* and *Pilidiella granati*^2^,^7^,^8^,^9^,^10^. However, a recent increase in the incidence of crown rot, dieback and twig blight caused by *P. granati* has been documented from pomegranate cultivation areas of various countries including China, Greece, Turkey, Iran, Spain Israel and Italy. The *P. granati* (Syn. *Coniella granati*) is an ascomycete that produces globose pycnidia with black thin pesudoparenchatmic walls. Single cell pycnidiospores overwinter in the dead shoots, fruit mummies, and prunings. These spores can spread by rain or water and cause latent infection to the surface of the young pomegranate fruits and trees^7^,^11^,^12^. In crown rot or dry rot, the fungal infection causes the necrosis that starts from the sepals and spread to the entire surface of the fruit causing its shriveling. Whereas, in the case of twig blight, the necrosis starts from the lower part of stem leading to wilting and dieback of the young branches and growing root suckers^7^,^12^,^13^,^14^,^15^,^16^.

China was ranked first in the world with 1.2 million tons annual production of pomegranate and total planting area about 120,000 hm^2^ in 2012^17^. *P. granati* has caused substantial economic loss to pomegranate industry in a number of countries including China^7^. We have previously reported *P. granati* as a casual agent of twig dieback and fruit rot with 10 and 30% disease incidence in the major pomegranate cultivation area of China^14^. The pathogen reduced both the quality and yield of pomegranate. Therefore, it is necessary to develop a rapid and accurate method for the detection of *P. granati* that can be implemented for the routine diagnosis and management of the pathogen.

Traditional fungal identification protocols include isolation, culturing and studying the morphological characters combined with physiological tests. These methods are labor intensive, time-consuming. Moreover, highly skilled and experienced personnel are required to identify less commonly encountered pathogens and variant strains^18^,^19^. However, with the advancement in the molecular biology, authentic DNA barcodes are available as a powerful tool for the identification of fungal species. One of the commonly used markers is highly repetitive internal transcribed spacer (ITS) sequences within the ribosomal RNA gene cluster. The success of these sequences along with PCR has eliminated the use of even more correct fungal protein-coding DNA sequences^18^,^19^,^20^,^21^,^22^.

PCR-based diagnostic methods are well documented for numerous plant pathogens, including bacteria, viruses, and fungi^23^,^24^,^25^. These methods are rapid, sensitive and highly specific^26^. Therefore, in present work, nested PCR technique has been used for the rapid and accurate detection of *P. granati* in pomegranate. Furthermore, this is the first report on the PCR-based approach to detect *P. granati*.

## Results

### Primer design and nested PCR

**Figure 1:**
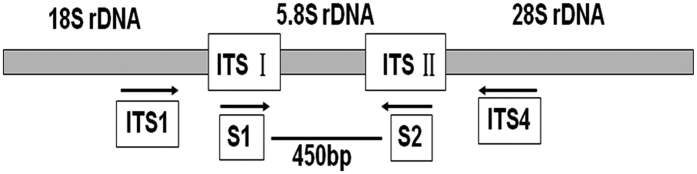
Illustration of positions of universal primers (ITS1 and ITS4) and specific primers (S1 and S2) in the ribosomal RNA gene cluster.

**Figure 2:**
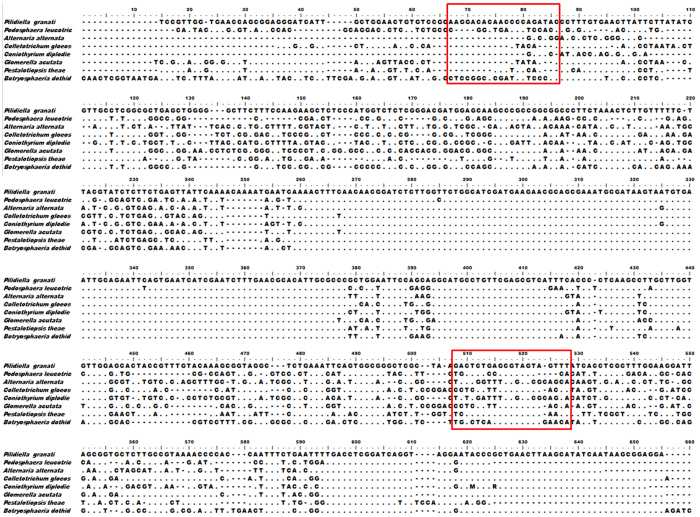
Alignment of partial sequences of ITS regions of rDNA of selected fungi. The red frame indicates the selected primers.

In the present work, the nested PCR method has been developed for the detection of *P. granati* in the pomegranate fruit. In order to design the specific primers, ITS sequence of 5.8S rDNA of *P. granati* (GenBank accession No. KF560320.1) was used (Fig. 1). The target sequence was compared with 5.8S ITS regions of seven other fungal strains (Table 1) using BioEdit v7.0.5 software. The aligned sequences were used to design the S1 and S2 primers (Fig. 2). In the first round of amplification, universal primer pair ITS1 ⁄ ITS4 was used. Whereas, in the second round of amplification, a predicted 450-bp DNA fragment was successfully amplified using S1 and S2 primers.

### Specificity of the assay

**Figure 3:**
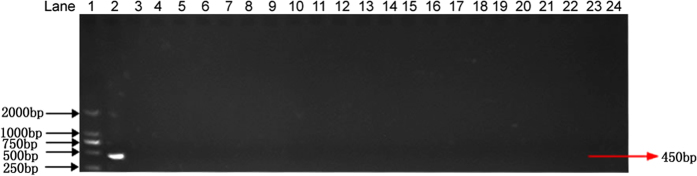
PCR for the detection of *Pilidiella granati* with S1 and S2 primers. Lane 1: DNA ladder; lane 2: Positive control (*Pilidiella granati*); lane 3: *Alternaria alternata*; lane 4: *Phomopsis fukushii*; lane 5: *Botryosphaeria dothidea*; lane 6: *Fusarium oxysporum*; lane 7: *Botrytis cinerea*; lane 8: *Ascochyta eriobotryae*; lane 9: *Coniothyrium diplodiella* (syn. of *Pilidiella diplodiella*); lane 10: *Pestalotiopsis clavispora*; lane 11: *Colletotrichum gloeosporioides*; lane 12: *Aspergillus flavus*; lane 13: *Podosphaera leucotricha*; lane 14: *Alternaria mali*; lane 15: *Phomopsis amygdalina*; lane 16: *Glomerella cingulata*; lane 17: *Gymnosporangium haraeanum*; lane 18: *Sclerotinia sclerotiorum*; lane 19: *Glomerella acutata*; lane 20: *Pestalotiopsis punicae*; lane 21: *Plasmopara viticola*; lane 22: *Pestalotiopsis theae*; lane 23: *Monilinia fructicola*; lane 24: negative control.

**Figure 4:**
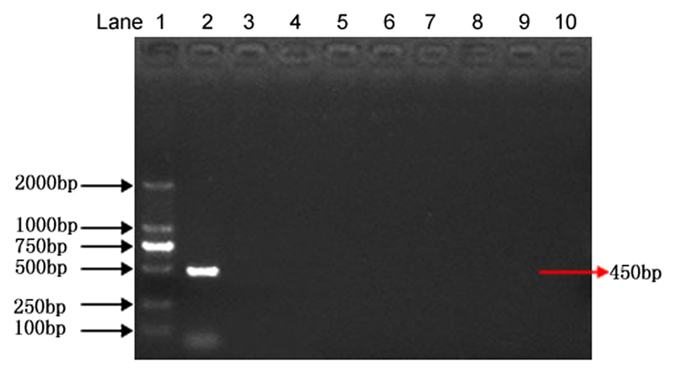
Nested PCR for the detection of pomegranate pathogens with S1 and S2 primers. Lane 1: DNA ladder; lane 2: *Pilidiella granati*; lane 3: *Glomerella cingulata*; lane 4: *Penicillium purpurogenum*; lane 5: *Botrytis cinerea*; lane 6: *Aspergillus niger*; lane 7: *Pestalotia brevista*; lane 8: *Alternaria* spp.; lane 9: *Trichoderma* spp.; lane 10: negative control.

The specificity of the primers was tested by using genomic DNAs of 21 different fungal pathogens (Table 2). An expected 450 bp DNA fragment was amplified using the S1/S2 primers only from *P. granati*. No PCR products were obtained from the other tested fungal strains (Fig. 3). The specificity was further tested by using the genomic DNA of five other fungal pathogens of pomegranate (*Glomerella cingulate, Penicillium purpurogenum, Monochaetia pachyspora, Cercospora punicae* and *Sphaceloma punicae*). Again, no PCR products were obtained with these pomegranate pathogens (Fig. 4). The amplification of PCR product exclusively from the genomic DNA of *P. granati* indicated that the designed primers were especially specific for the target pathogen.

### Sensitivity of the assay

**Figure 5:**
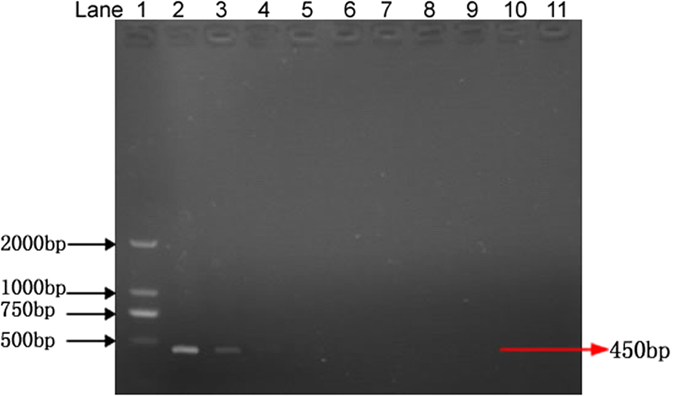
Sensitivity of the conventional PCR using S1 and S2 primer pair for the detection of *Pilidiella granati.* Lane 1: DNA ladder, lane 2–10: template DNA concentrations (100 ng, 10 ng, 1 ng, 100 pg, 10 pg, 1 pg, 100 fg, 10 fg, 1 fg, respectively); lane 11: negative control

**Figure 6:**
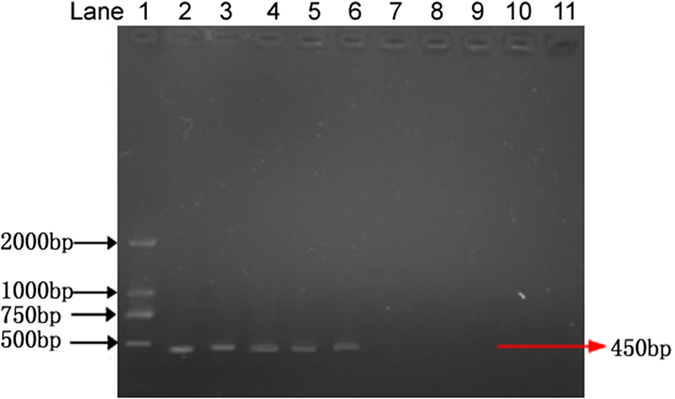
Nested PCR for the detection of *P. granati.* Lane 1: DNA ladder; lane 2–10: template concentrations (100 ng, 10 ng, 1 ng, 100 pg, 10 pg, 1 pg, 100 fg, 10 fg, 1 fg, respectively); lane 11: negative control.

The sensitivity of the designed protocol was tested by using different concentrations of genomic DNA of *P. granati* as a template in the individual nested PCR assays. In the first step, the conventional PCR reaction was carried out using S1 and S2 primers. The PCR product analysis indicated that the lower limit for the detection of target pathogen was 10 ng of DNA per 25 μl of PCR mixture (Fig. 5). To increase sensitivity, the nested PCR protocol was performed using a universal primer pair (ITS1 and ITS4) and a primary PCR primer pair (S1 and S2). This enhanced the sensitivity of the assay and the detection of the pathogen with 10 pg of DNA was obtained (Fig. 6). Thus, nested PCR increased the lower detection limit of genomic DNA from 10 ng to 10 pg.

### Detection of *P. granati* in pomegranate fruit

**Figure 7:**
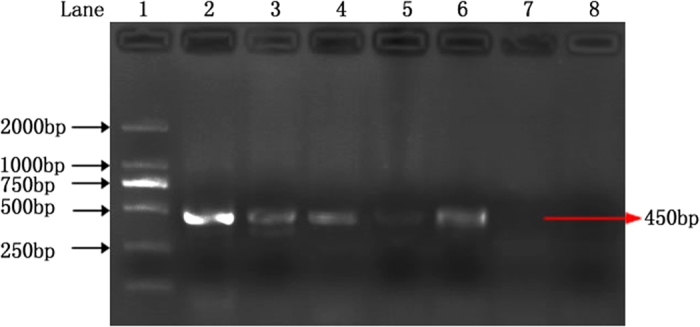
The Nested PCR assay as diagnostic test for the detection of *Pilidiella granati* in the pomegranate fruits. Lane 1: DNA ladder; lane 2: positive control (genomic DNA *of P. granati*); lane 3–4: naturally infected samples; lane5-6: artificially infected samples; lane 7: Control (healthy) sample; lane 8: negative control.

The nested PCR was performed to diagnose the *P. granati* infection in the pomegranate samples that were collected from the different areas of Anhui Province, China. To validate the protocol, artificially infected pomegranate fruits were also used. The genomic DNAs were isolated from naturally infected, artificially infected and healthy control fruits and subjected to the nested PCR assay. Both the naturally infected and artificially infected samples were found to be positive for *P. granati* as a 450-bp PCR product was obtained on the agarose gel. Whereas, no PCR products were obtained with DNA from the control samples (Fig. 7).

## Discussion

The disease caused by *P. granati*, is an emerging threat to the rapidly expanding pomegranate industry in many regions of the world. It has been reported to cause crown rot, dry rot and dieback twig blight of pomegranate in many countries including Eastern Mediterranean, Turkey, India, Greece, Cyprus, and China^7^,^9^,^12^,^14^,^15^,^16^. A comprehensive survey in Greece showed that disease incidence was 29 and 50% of pomegranate fruit rot by *P. granati* at various locations in 2011 & 2012, respectively that increased to 34–53% in all the commercial pomegranate orchards in 2014. Pycnidia of the pathogen were found in 77% of the mummified fruits, 25% of the blighted shoot and 19% of the crown of trees with symptoms of rots that were left in the orchard. Moreover, the disease incidence was higher in the areas where dark brown to black fruit mummies were seen scattered on the orchard floor^7^. In a few countries, the pomegranate disease caused by *P. granati* has already acquired the status of quarantine disease. In 2006, all the grafting material that imported from India was destroyed after the diagnosis of *C. granati* in Israel^15^.

To develop active surveillance and management of dry rot in pomegranate industry is critical for avoiding the yield losses by *P. granati*.^27^. A rapid and precise detection of *P. granati* is a preliminary step to achieve this goal. However, traditonal identification appraoch involves the identification based on culturing and morphology, which is time consuming^18^.

Molecular-based methods such as PCR have greatly improved the detection of microbes present in the environment^28^. PCR based assays are more rapid, sensitive, specific and accurate and have been often implemented for the routine diagnostics of a variety of pathogens^24^,^25^,^29^,^30^,^31^,^32^,^33^. In the present work, we have used nested PCR as a rapid approach for the detection of *P. granati*. Analysis of ITS sequences of rDNA of *P. granati* and seven other fungal strains was performed to design primary PCR primer pair. The developed protocol was successfully used for the exclusive amplification of the 450 bp fragment from *P. granati* genomic DNA. Thus, this method can discriminate *P. granati* from all the other fungi tested. In the consortia of the barcodes of life, ITS sequences of nuclear rDNA serve as universal DNA barcodes. These loci have become very attractive alternatives to the traditional protocols mainly due to the development of successful PCR and sequencing methods. Even though the ITS sequences can be readily amplified by universal ITS primers, there is still sufficient interspecific sequence divergence. This diversity within ITS region can be exploited for the species identification by using carefully designed species-specific primers^18^,^22^,^24^,^34^. Therefore, in the present work ITS region of the *P. granati* was used to develop the detection protocol.

The primer with high specificity in the PCR based diagnostics is of prime importance. Therefore, 21 different fungal strains, including *P. diplodiella* were used to test the specificity of the S1/S2 primer pair. In the second round of amplification, no PCR products were obtained with any of the tested strains. Only *P. granati* gave the positive results. The specificity of the designed primers was also tested for the seven different pomegranate pathogens. However, again, no PCR products were obtained with any of these pathogens. Thus, these results indicate that the developed protocol is specific for the *P. granati*. The primers (S1 and S2) designed in the present nested PCR protocol are not claimed to be highly species specific. Even though, when the designed primer pair was used to detect *P. diplodiella*, no PCR products were obtained. We did not aim to make the primers highly species specific because no other *Pilidiella* species have been reported to infect pomegranate plant. *P. granati* is host specific and the sole pathogen of the pomegranate from the genus *Pilidiella*. When it infects the pomegranate, it penetrates inside the host tissues. Thus, host tissues might be used for detection of the pathogen. Moreover, in the developed protocol, the samples were surface sterilized before the extraction of fungal genomic DNA. Consequently, the probability of the presence of any other *Pilidiella* species as a contaminant inside the fruit tissues is very rare. Therefore, no further work was carried out to analyze and improve the species-specificity.

Although the conventional PCR is considered to be the most suitable diagnostic technique for the detection of various kinds of pathogens. It has certain detection limit when the target DNA concentration is low. It is very often necessary to enhance the sensitivity of the reaction. Several PCR techniques, notably including nested PCR, qPCR, Bio-PCR and co-operational PCR coupled with dot blot hybridization, have been developed to increase the sensitivity of the PCR based assays. Among these, nested PCR is the most frequently used method to obtain the acceptable level of sensitivity^19^,^24^,^28^,^35^,^36^,^37^. The earlier infection of *P. granati* in the pomegranate plants and young fruits is either latent or too low to be detected. In the present work, when conventional PCR was used, the lower detection limit for template DNA was 10 ng. The nested PCR technique was used to enhance the sensitivity of the PCR assay. This increased the sensitivity of the assay and detection of the pathogen was possible when as low as 10 pg of *P. granati* DNA was present. Many other researchers have used nested PCR to increase the sensitivity of the reaction for the detection of pathogens^19^,^24^,^37^,^38^,^39^,^40^,^41^,^42^.

To validate the current protocol, healthy pomegranate fruits were artificially inoculated with *P. granati* followed by the detection of pathogen. The genomic DNA was extracted from the artificially inoculated, naturally infected and control healthy samples followed by detection of the pathogen by nested PCR approach. The results showed that the developed protocol successfully detected the *P. granati* infection only in both the naturally and artificially infected pomegranate fruit in 6 h. No PCR products were obtained in healthy samples. Thus, these results indicate that method developed in the present work is rapid, accurate and highly sensitive. It is a promising and alternative method to the traditional diagnostic and identification protocols for the detection of *P. granati*. This method will be useful for the early detection of *P. granati* infection. The technique will be helpful, especially for the farmers to manage the disease in time. Furthermore, this method can also be applied to study the epidemic trends of this disease in the pomegranate cultivation regions.

## Methodology

### Fungal strains

All the fungal strains used in this work were isolated from the different fruits. These fruits were collected from the different areas of Anhui Province, China. These fungal cultures were maintained on the potato dextrose agar (PDA) medium and stored at 4 °C. The isolates were firstly identified by cultural and morphological characters. The identity of these strains was further confirmed by PCR using ITS1 and ITS4 universal primers followed by standard sequencing. The sequences were used to identify the isolates by using the online bioinformatic tool BLASTN^43^.

### Extraction of fungal genomic DNA

Fungal strains were grown on the individual PDA plates at 28 °C for 48–72 h. The fungal mycelial mass (50 mg) from each strain was used to extract genomic DNA using the Fungal DNA Kit (Omega Bio-Tek). The isolation was carried out according to the manufacturer’s protocol. DNA concentration for each sample was measured by using NanDrop UV spectrophotometer (NanoVue Plus, GE Healthcare Life Sciences).

### Primer designing

The primers were designed using ITS sequence of *P. granati* (GenBank accession No. KF560320.1). The target sequence was compared with that from eight different fungal species including *P. granati* (Table 1) by using software BioEdit v7.0.5. Forward and reverse primers i.e. S1: 5′-AAGGACACAACCCCAGATAC-3′ and S2:5′-ATAAACTACTACGCTCAGAG-3′, were designed to amplify 5.8S ITS region of rDNA of *P. granati* (Fig. 1). These primers were used for the second round of amplification during nested PCR.

### Nested PCR

First round of nested PCR was carried out using universal primers ITS1 (5’-TCCGTAGGT GAACCTGCGG-3′) and ITS4 (5′-TCCTCCGCTTATTGATATGC-3′)^44^. The amplification was performed in PCR tube containing 10X Taq buffer (2.5 μl), 25 mM MgCl_2_ (2.0 μL), 0.8 mM dNTP, 0.4 μm of each of ITS1 and ITS4 primers, 5 U Taq DNA polymerase and 50 ng template DNA. The final volume of the reaction mixture was made up to 25 μL with sterile distilled water. The optimized thermocycler conditions for the reaction were initial denaturation at 94 °C for 5 min, 35 cycles at 94 °C for 30 s, 58 °C for 30 s, 72 °C for 30 s and final extension at 72 °C for 10 min. The second round of amplification was carried out using same final concentration of the reagents as described above, except replacing the DNA template with 0.5 μl PCR product from the first round of amplification. The thermocycler conditions were also kept the same except that the annealing temperature was reduced to 52 °C. The PCR products were checked using 1% agarose gel with DNA ladder DL2000.

### Specificity of the assay

Specificity of the S1 and S2 primer pair for the detection of *P. granati* was determined by using the genomic DNAs isolated from *P. granati* and 21 different fungal species (Table 2). The genomic DNAs isolated from these strains were used as template for the nested PCR assay as described above. To confirm the specificity of the primers for different pomegranate pathogens, the nested PCR assay was carried out using the seven common pomegranate pathogens including *Glomerella cingulate, Penicillium purpurogenum, Botrytis cinerea, Aspergillus niger, Alternaria* spp., *Trichoderma* spp., *Pestalotia brevista*.

### Sensitivity of the assay

The sensitivity of the nested PCR for the detection of *P. granati* was determined by using the different concentrations (1.0 ng–100 fg) of genomic DNA as template.

### Detection of *P. granati* in the infected fruits

The healthy and infected fruit samples were collected from the different orchards of Huaiyuan County, Anhui, China in sterile polythene bags and stored at 4 °C in laboratory conditions. The artificially infected samples were prepared by inoculating the healthy fruits with *P. granati*^14^. The genomic DNA was isolated from the artificially inoculated, naturally infected and healthy (control) pomegranate samples by using the standard protocol^45^ with minor modification. The surface of each sample was disinfected with 75% ethanol for 1 min and washed with sterile water twice. About 50 mg of each fresh fruit tissues was individually grounded in liquid nitrogen with a twister in a 1.5 mL Eppendorf tube. After that 900 μl CTAB extraction buffer and 90 μl SDS (10%, w/v) were added to the each tube and vortexed. The tubes were incubated at 60 °C for 1 h. The genomic DNA was extracted from the supernatant with phenol/trichloromethane/isoamyl alcohol mixture (25:24:1) followed by precipitation with equal volume of isopropanol. The pellet was washed twice with 70% ethanol. The pellet was air dried and dissolved in 70 μl TE buffer. The DNA concentration of each sample was estimated by the Nanodrop UV spectrophotometer (NanoVue Plus, GE Healthcare Life Sciences). The nested PCR was performed as described above. The genomic DNA from the *P. granati* was used as positive control in all the experiments. In negative control, genomic DNA was replaced with sterile distilled water.

## Additional Information

**How to cite this article**: Yang, X. *et al*. Development of a nested-PCR assay for the rapid detection of *Pilidiella granati* in pomegranate fruit. *Sci. Rep.* **7**, 40954; doi: 10.1038/srep40954 (2017).

**Publisher's note:** Springer Nature remains neutral with regard to jurisdictional claims in published maps and institutional affiliations.

## Figures and Tables

**Table 1:** List of fungal species and their hosts used for the primer design.

Sr. No.	Fungal species	GenBank Accession No.	Host plant
1.	*Pilidiella granati*	KF560320.1	Pomegranate
2.	*Alternaria alternata*	JQ625589.1	tomato
3.	*Botryosphaeria dothidea*	JF800138.1	apple
4.	*Pilidiella diplodiella* (Syn. of *Coniothyrium diplodiella*)	EU520203.1	grape
5.	*Colletotrichum gloeosporioides*	KP748204.1	pepper
6.	*Podosphaera leucotricha*	HM579838.1	peach
7.	*Glomerella acutata*	FN566876.1	orange
8.	*Pestalotiopsis theae*	JN943624.1	tea

**Table 2:** List of fungal species and their hosts used to test primer specificity.

Sr. No.	Fungal species	Host plant
1.	*Pilidiella diplodiella*	grape
2.	*Alternaria alternata*	pear
3.	*Alternaria malii*	apple
4.	*Ascochyta eriobotryae*	loquat
5.	*Aspergillus flavus*	pear
6.	*Botryosphaeria dothidea*	pear
7.	*Botrytis cinerea*	peach
8.	*Colletotrichum gloeosporioides*	apple
9.	*Fusarium oxysporum*	strawberry
10.	*Glomerella acutata*	nectarine
11.	*Glomerella cingulate*	pomegranate
12.	*Gymnosporangium haraeanum*	pear
13.	*Monilinia fructicola*	peach
14.	*Pestalotiopsis punicae*	pomegranate
15.	*Pestalotiopsis theae*	loquate
16.	*Pestalotiopsis clavispora*	blueberry
17.	*Phomopsis amygdalina*	peach
18.	*Phomopsis fukushii*	pear
19.	*Plasmopara viticola*	grape
20.	*Podosphaera leucotricha*	strawberry
21.	*Sclerotinia sclerotiorum*	pear
